# High-tolerance crystalline hydrogels formed from self-assembling cyclic dipeptide

**DOI:** 10.3762/bjnano.10.184

**Published:** 2019-09-18

**Authors:** Yongcai You, Ruirui Xing, Qianli Zou, Feng Shi, Xuehai Yan

**Affiliations:** 1 Beijing Advanced Innovation Center for Soft Matter Science and Engineering & State Key Laboratory of Chemical Resource Engineering, Beijing University of Chemical Technology, Beijing, 100029, China; 2 State Key Laboratory of Biochemical Engineering, Institute of Process Engineering, Chinese Academy of Sciences, Beijing 100190, China; 3 University of Chinese Academy of Sciences, Beijing 100049, China

**Keywords:** crystalline hydrogel, cyclic dipeptide, electrochemical supercapacitors, nanoarchitectonics, self-assembly

## Abstract

Peptide-based supramolecular hydrogels, as a new type of biological nanoarchitectonic structure, hold great promise for a wide range of biomedical and nanotechnological applications, such as tissue engineering, drug delivery, and electronic and photonic energy storage. In this work, a cyclic dipeptide (CDP) cyclo-(Trp-Tyr) (C-WY), which has exceptional structural rigidity and high stability, is selected as a hydrogelator for the formation of supramolecular hydrogels. The unique hydrogen bonding in C-WY endows a high propensity for self-assembly and the resulting hydrogels are revealed to be crystalline. The crystalline hydrogels possess excellent mechanical capacity and superior tolerance to various harsh conditions, including in the presence of charged biopolymers, extreme acid/base environments, and changing thermal conditions. Such high tolerance enables the crystalline hydrogels to be applied in the complex and harsh environments of electrochemistry. In addition, this study demonstrates that the self-assembly of cyclic dipeptides results in highly robust hydrogels which can be applied for electrochemical applications such as electrochemical supercapacitors.

## Introduction

On account of their high water content and highly tunable mechanical properties, hydrogels as soft nanoarchitectonics and soft matter are well-suited in extensive applications, such as tissue engineering, drug delivery, and electronic and photonic energy storage [1–10]. Self-assembled peptide materials have shown outstanding characteristics, such as excellent biocompatibility, structural flexibility, versatile functionality, and low immunogenicity [11–28]. Peptides can be deliberately engineered to self-assemble into well-ordered hydrogels with adjustable mechanical and physicochemical properties [29–33]. Peptide-based supramolecular hydrogels have been widely used in biological and nanotechnology fields [34]. However, linear peptide-based hydrogels usually have several deficiencies, such as poor molecular rigidity, disabled mechanical modulus (storage or loss modulus), and poor environmental tolerance under thermal, acidic, or alkaline conditions [35–37]. Hence, new types of peptide hydrogels are highly needed to promote the practical applications of peptide hydrogels.

Cyclic dipeptides (CDPs), which are based on the basic structures of heterocyclic 2,5-diketopiperazines, are a special kind of dipeptides. They are the smallest cyclic peptides and contain six-membered heterocyclic lactam ring cores. CDPs exhibit exceptional structural rigidity, stability, as well as biological activity as compared to their linear counterparts [38–41]. There are many natural CDPs since they can be produced as secondary metabolites in many organisms. Hence, these are ideal raw materials for engineering functional architectures because of their unique biosecurity. Especially, CDPs contain four hydrogen-bonding sites, which provide a substantial tendency for self-assembly and the formation of gels. In addition, other weak forces, such as π–π stacking, hydrophobic effect, electrostatic interactions, and van der Waals forces, are also serviceable in driving molecular self-assembly of CDPs toward the formation of gels.

Gels prepared by CDP self-assembly integrate the advantages of low molecular weight gels, including the multiple functionalities, adjustable performance, and dynamic features. CDP-based gels have been developed as smart soft materials for a multitude of applications. However, most of the attention is focused on the amorphous assemblies in organic solvents and ionic liquids [42–46]. Although these CDP gels have good mechanical properties and deceased enzymatic degradation under physiological conditions, they still have some challenging problems such as inflexibility, low biosecurity and precipitation formation [47].

**Scheme 1:**
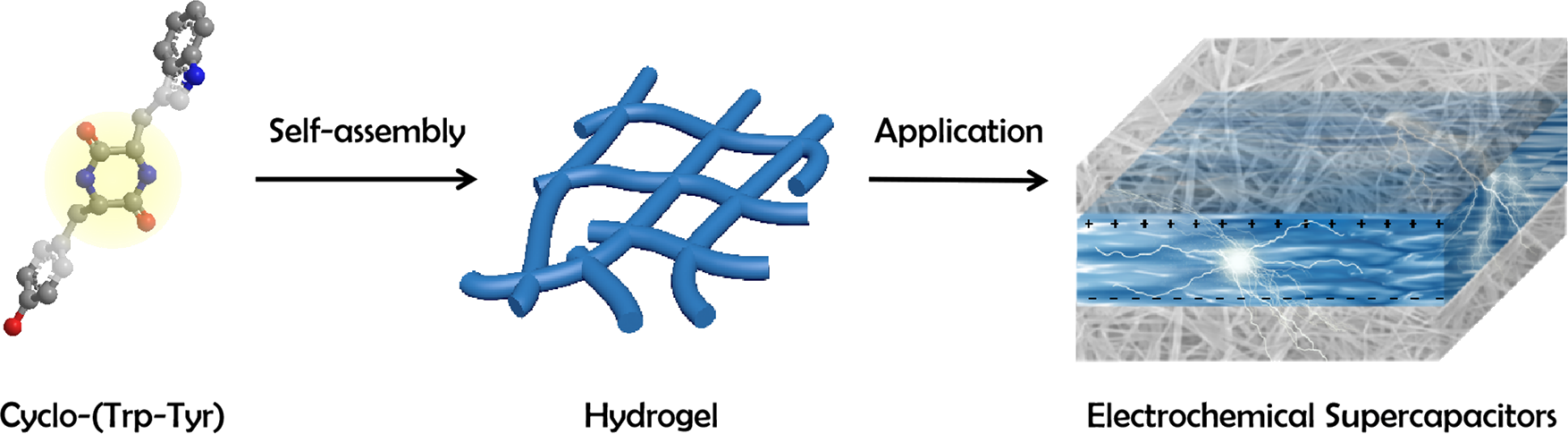
Schematic of the formation of self-assembled C-WY hydrogels and their applications in electrochemical supercapacitors.

Herein, we investigate the self-assembly and application of a CDP, cyclo-(Trp-Tyr) (C-WY) (Scheme 1). C-WY contains a rigid six-member ring as a bridge, which increases the structural rigidity and stability. The abundant hydrogen bonds in C-WY endow a high propensity for self-assembly. In the previous example, the feasibility of C-WY peptide nanotubes as carriers of caspase 3 to silence shRNA delivery was verified. Based on these excellent characteristics of C-WY, it was selected as a hydrogelator for the formation of supramolecular hydrogels. The self-assembly of C-WY forms a hydrogel with crystal features and close-knit three-dimensional network structures. Importantly, the C-WY hydrogel exhibited adjustable rheological properties, excellent stability, and high tolerance under various conditions, including in the presence of charged biopolymers (poly-ʟ-lysine (PLL), alginate (ALG), hyaluronic acid (HA)), extreme acid/base environments, and thermal conditions. Owing to the robustness of the hydrogel, the material also showed excellent performance as an electrochemical supercapacitor. Hence, self-assembled CDP hydrogels are promising for applications in complex and harsh environments.

## Results and Discussion

### Preparation and characterization of the hydrogel

**Figure 1:**
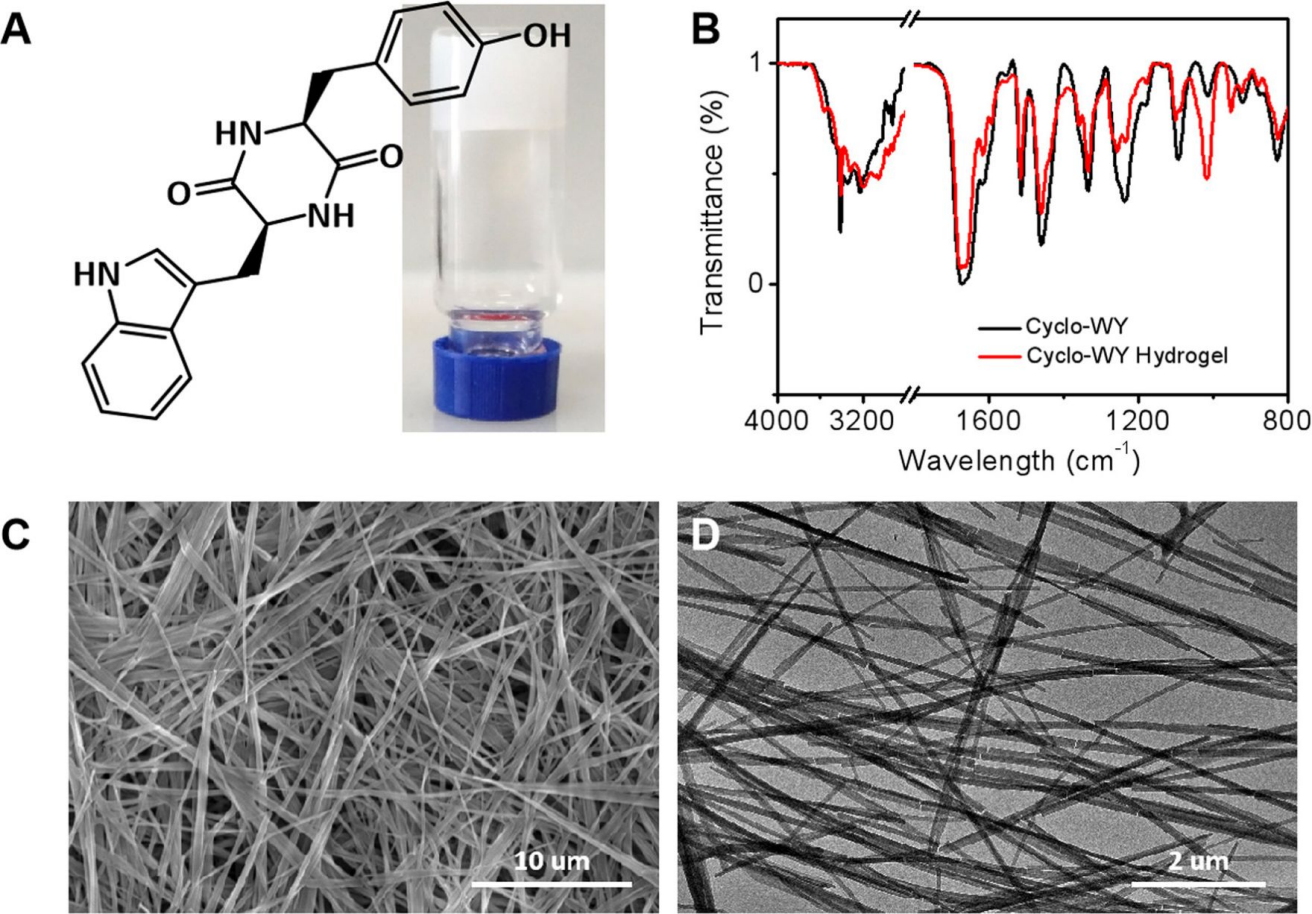
CDP-based supramolecular hydrogels. (A) The structure of C-WY and a photo of the C-WY hydrogel. (B) FTIR spectra of C-WY powder and the C-WY hydrogel. (C) SEM and (D) TEM images of the C-WY hydrogel.

C-WY was chosen as a model peptide to investigate the self-assembly of CDPs. A nontransparent hydrogel with a dense network of fibers was obtained simply by mixing a solution of C-WY in DMSO (2 mg, 20 μL) with water (480 μL) (Figure 1A). The hydrogen bonding interactions between the C-WY molecules were investigated by Fourier-transform infrared spectroscopy (FTIR). Compared with the peak of amide N–H stretching located at 3344 cm^−1^ of the unimolecular C-WY, the hydrogel has a red-shifted amide N–H stretching band located at 3317 cm^−1^, indicating the formation of strong hydrogen bonds between C-WY molecules in the hydrogel (Figure 1B). Further characterization by scanning electron microscopy (SEM) and transmission electron microscopy (TEM) was performed to inspect the morphology of the hydrogel (Figure 1C,D). The fibers in the hydrogel are 100 ± 50 nm in width and dozens of micrometers in length. In addition, dense three-dimensional fibrous networks cross-linked by slender fibers were clearly observed. The cross-linked networks are beneficial to improving the stability of the hydrogels at extreme conditions [48]. The cross-linked networks are also the foundation for a range of biomedical and nanotechnological applications.

### Interior structure and crystal pattern

**Figure 2:**
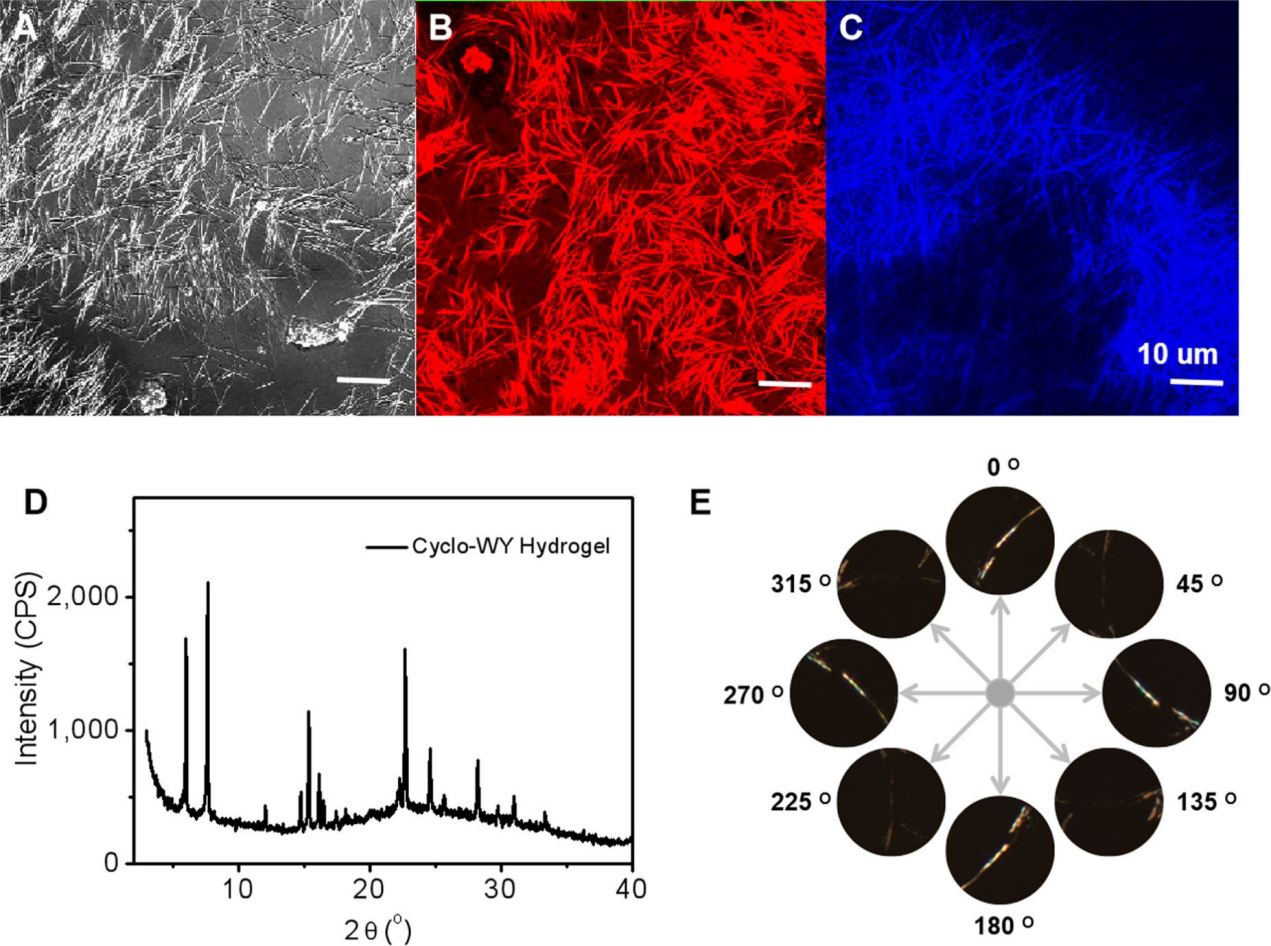
Interior structure and crystal pattern. (A) CLSM images of the C-WY hydrogel in light field. NR was used to indicate the formation of hydrophobic domains (red color, B) and ThT was used to indicate the beta-sheet secondary structures (blue color, C). (D) XRD pattern of the hydrogel. (E) POM images in cross-polarized light mode of samples taken at 0–360°.

The fibrillar structure and three-dimensional fibrous network of the C-WY hydrogel were further investigated by confocal laser scanning microscopy (CLSM) (Figure 2A). Thioflavin T (ThT) and nile red (NR), two specific dyes for hydrophobic domains and beta-sheet secondary structures, respectively, were used to obtain insights into the detailed interior structure of the hydrogel [49]. CLSM results confirmed that the C-WY hydrogel contains both hydrophobic domains (red regions) (Figure 2B) and beta-sheet secondary structures (blue regions) (Figure 2C). Intriguingly, the X-ray diffraction (XRD) results showed the presence of sharp peaks, indicating that the hydrogel has long-range, ordered, crystal patterns (Figure 2D). The crystal patterns were further confirmed by polarized optical microscopy (POM). POM images in cross-polarized light mode of a randomly selected fiber were taken ranging from 0° to 360° (Figure 2E). When the selected fiber was observed under a cross-polarized angle of 0°, the sample was bright. In contrast, the sample turned dark when the cross-polarized angle was changed to 45°. The changing contrast behaviors between dark and bright changed periodically along with the angle changing by 45°. These results illustrate that the fibers in the hydrogel are intrinsically crystalline and thus have polarization properties.

### Rheological properties

**Figure 3:**
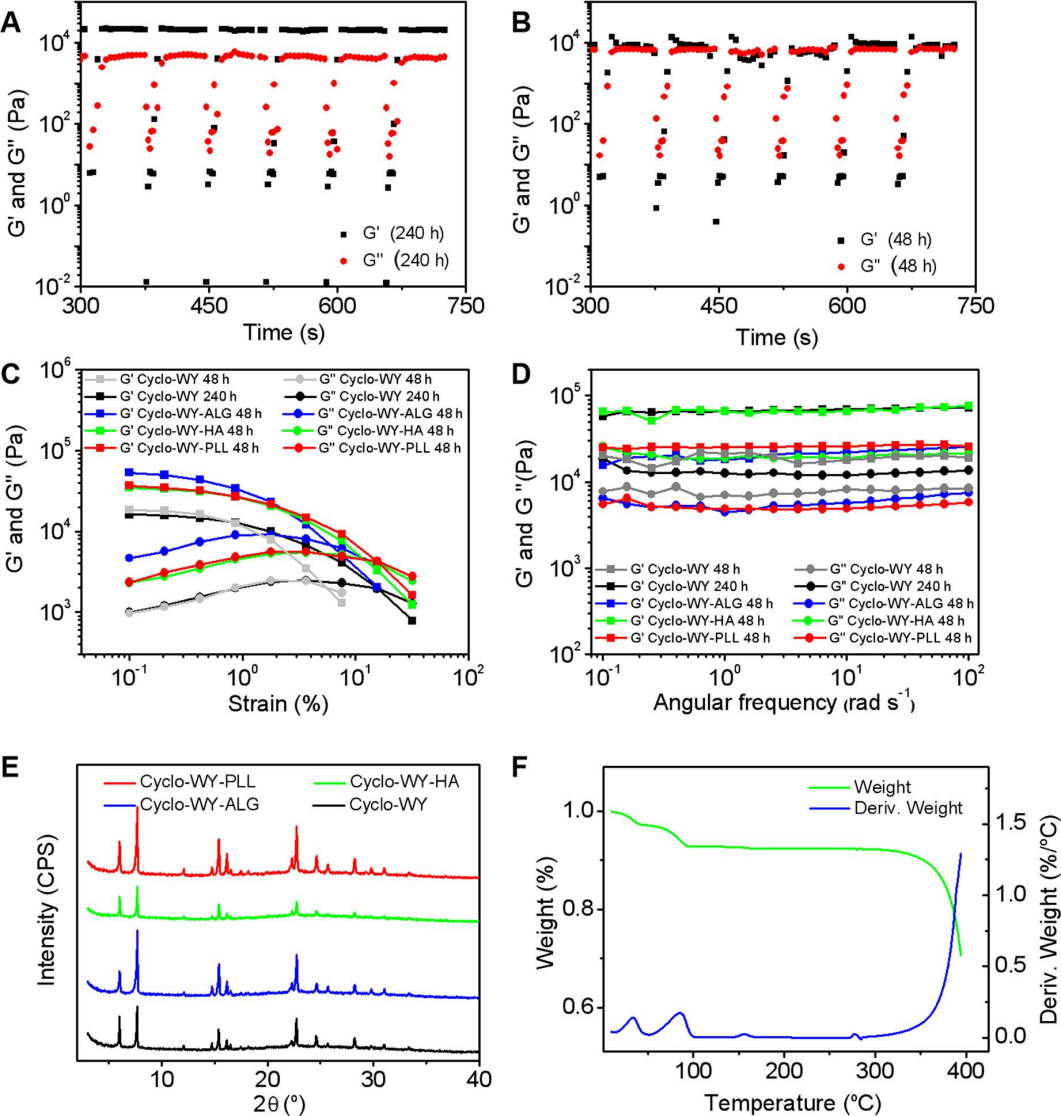
Rheological characterization and environmental tolerance. The self-healing capacity of the hydrogels at 48 h (A) and 240 h (B) demonstrated by the continuous step–strain experiments. Strain-dependent (C) and frequency-dependent (D) oscillatory shear rheology of the hydrogels under various conditions. (E) XRD patterns of hydrogels with various polymers. (F) TG curves of the C-WY hydrogel.

The rheological properties of hydrogels are key evaluation indicators for a variety of applications [50–51]. It is typically challenging for hydrogels based on linear peptides to maintain their original gel state for a long time or under shear force. Driven by a thermodynamic process, they tend to gradually form crystalline precipitations [34]. In order to investigate the rheological properties of the C-WY hydrogel, the storage (elastic, G’) and loss (viscous, G’’) modulus of the hydrogels aged for 48 h and 240 h were studied. The results showed that the mechanical capacity of the C-WY hydrogel enhanced along with time. Strain-induced shear-thinning and self-healing abilities of the hydrogel were detected through continuous step changes of oscillatory strain between 500% and 1% (at a constant frequency of 1 rad s^−1^). Under a high magnitude strain (500%), the modulus of G’’ values exceeded G’ values, indicating the breaking of the hydrogel (Figure 3A). By decreasing the strain to 1%, the modulus of G’’ falls below that of G’, indicating the recovery of the hydrogel (Figure 3B). These results illustrated that the recovery of the hydrogel is quick at both 48 h and 240 h, even at the fifth test cycle. As compared to that of 48 h, the C-WY hydrogel at 240 h showed a faster recovery speed. The strain-dependent oscillatory rheology results (at 240 h) showed a great anti-shear performance at stains ranging from about 0.1% to about 20%, indicating the shear-thinning behavior of the hydrogels. The hydrogel at 48 h was broken at a strain of more than 6% (Figure 3C). The hydrogels at both 48 h and 240 h exhibited broad linear viscoelastic regions ranging from 0.1–100 rad s^−1^ in frequency-dependent oscillatory shear rheology experiments (at a constant strain of 1%, Figure 3D). Meanwhile, the modulus (both G’ and G’’) was enhanced after aging for a longer time, indicating the improvement in the mechanical capacity of the hydrogels with time. Taken together, the rheological study indicates that the CWY hydrogels possess shear-thinning and self-healing behaviors, which are time-dependent and important for their applications.

### Environmental tolerance

CDPs usually exhibit superior physical, chemical and thermal stability compared to their linear counterparts [52]. The hydrogels assembled from CDPs are therefore highly promising for practical applications. In order to study the stability of the C-WY hydrogel, charged biopolymers, including positively charged PLL and negatively charged HA and ALG, were selected for co-incubation with the hydrogel. The mechanical properties, including modulus (G’, G’’), shear-thinning behavior, and self-healing capability, improved after the introduction of the biopolymers whether the biopolymers were positively or negatively charged (Figure 3C,D). Clearly, the electrostatic repulsion or attraction between the C-WY and biopolymers contributes to the enhancement of intermolecular interactions in the hydrogel, leading to the improvement of the rheological properties. The crystal structure remained unchanged (Figure 3E) and no obvious aggregation or precipitation was observed. Also, the microtopography of the hydrogel exhibited no obvious changes (Supporting Information File 1, Figure S1).

The environmental conditions, such as pH and temperature, play pivotal roles in practical application [53]. Also, the pH or temperature is closely related to the intermolecular forces in nanomaterials and thus always affects the stability of hydrogels. The morphology and mechanical properties of the C-WY hydrogels showed no significant change when they were incubated in acid or base solutions (pH 1 or pH 14) for 24 h (Supporting Information File 1, Figure S2 and S3). In addition, the thermogravimetric (TG) curves verified the robustness of the hydrogels for resisting temperatures ranging from 0 °C to about 350 °C. The TG curves of the hydrogels exhibited three thermal decomposition steps (Figure 3F). The first two temperature points were ≈40 °C and ≈95 °C, which should be mainly assigned to the loss of water. The third point was ≈350 °C, which should be assigned to the decomposition of C-WY molecules. The tolerance of hydrogels towards resisting biopolymers, pH and heat, may be due to the entangled fiber network and extensive intermolecular hydrogen bonds in the hydrogel. In summary, the C-WY hydrogel was found to exhibit excellent environmental tolerance, including resistance to biopolymers, pH, and heat. Hence, the C-WY hydrogel is promising for applications in harsh environments, such as those of electrochemical supercapacitors.

### Application in electrochemical supercapacitors

**Figure 4:**
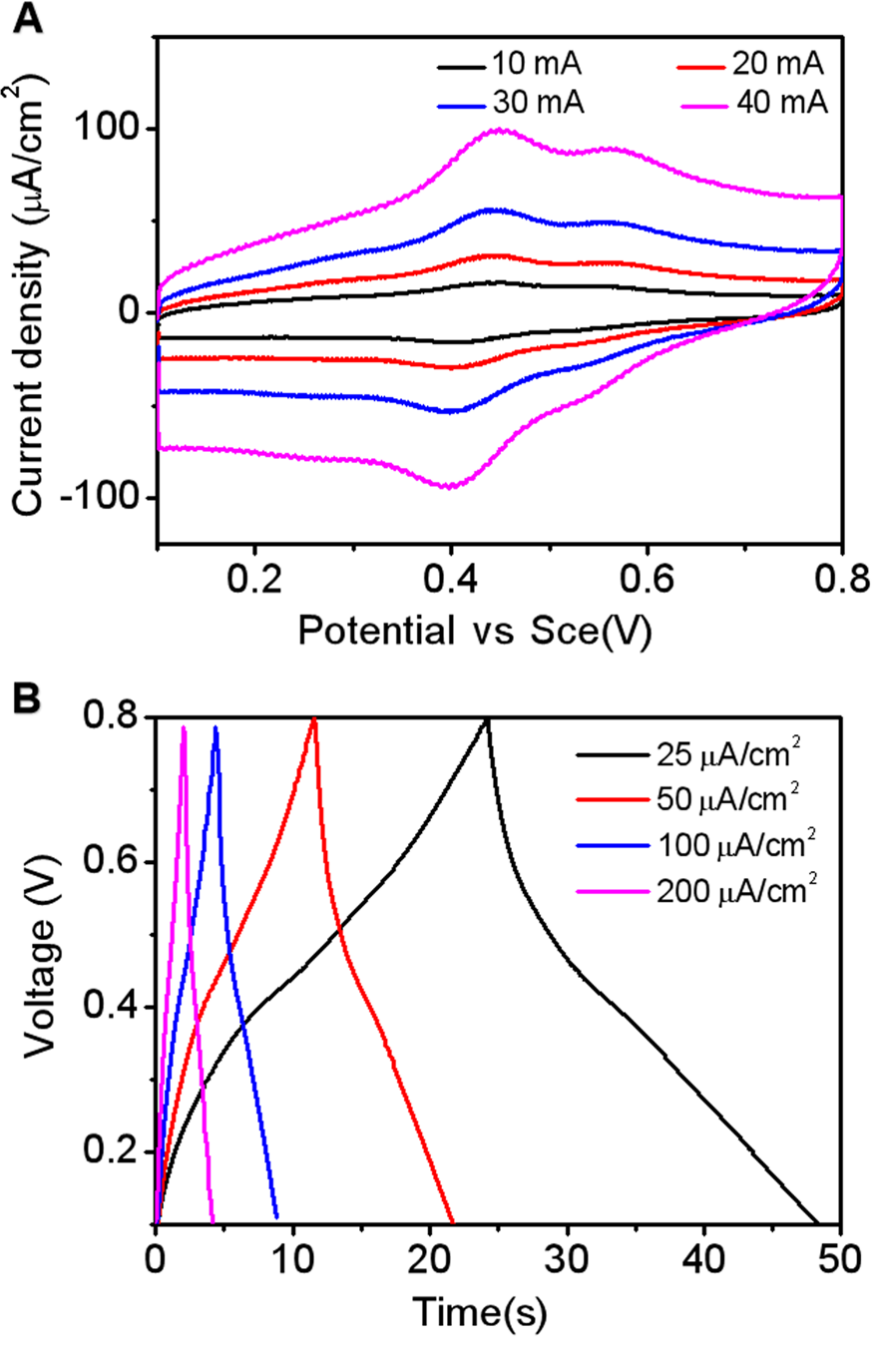
Characterization of hydrogels as supercapacitors. (A) Cyclic voltammograms at different scan rates. (B) Galvanostatic charge–discharge curves of C-WY hydrogels at different current densities.

Inspired by the high stability in harsh environments, we next investigated the application of the C-WY hydrogel as a candidate material for electrochemical supercapacitors. Constraint peptides are known for their superior chemical and stability compared to their linear counterparts. The hydrogels assembled from constraint C-WY peptides are therefore highly promising for application in bio-nanotechnology owing to their excellent stability from long-range ordered packing. Cyclic voltammetry (CV) curves of the hydrogel at different scan rates ranging from 10 to 40 mV were studied (Figure 4A). Typical capacitor shapes were observed in the curves, indicating that the C-WY hydrogel can be applied for electrochemical supercapacitors. In addition, the capacitive charge–discharge curves with galvanostatic current densities ranging from 25 to 200 mA/cm^2^ showed that the hydrogel has excellent electrochemical stability (Figure 4B). These results indicate that hydrogels based on CDPs have commendable stability and thus can be applied as candidate components for supercapacitors.

## Conclusion

In summary, we demonstrated that C-WY peptides can self-assemble into well-ordered fibrous networks based mainly on the inherent intermolecular hydrogen-bonding interactions. The hydrogel has a crystalline structure, excellent rheological properties, superior stability, and ideal robustness under various conditions, such as acidic or basic environments, or in the presence of charged biopolymers. Given its excellent electrochemical stability, the highly stable hydrogel was successfully demonstrated as an electrochemical supercapacitor. This study demonstrates that hydrogels based on self-assembly of CDPs can be valuable candidates for applications in harsh environments.

## Supporting Information

File 1 Experimental section and additional figures.
