# Supplementary material for: High-tolerance crystalline hydrogels formed from self-assembling cyclic dipeptide
Source: Beilstein J Nanotechnol. 2019 Sep 18;10:1894–901. doi: 10.3762/bjnano.10.184 (PMC6774068; doi:10.3762/bjnano.10.184)
Supplement: File 1 — Experimental section and additional figures. [file Beilstein_J_Nanotechnol-10-1894-s001.pdf]

BEILSTEIN JOURNAL OF NANOTECHNOLOGY

Supporting Information

for

High-tolerance crystalline hydrogels formed from self-assembling cyclic dipeptide

Yongcai You, Ruirui Xing, Qianli Zou, Feng Shi and Xuehai Yan

Beilstein J. Nanotechnol. **2019,** *10,* 1894–1901. doi:10.3762/bjnano.10.184

Experimental section and additional figures

License and Terms: This is a supporting information file under the terms of the Creative Commons Attribution License (http://creativecommons.org/licenses/by/4.0). Please note that the reuse, redistribution and reproduction in particular requires that the authors and source are credited.
The license is subject to the *Beilstein Journal of Nanotechnology* terms and conditions: (https://www.beilstein-journals.org/bjnano)

Experimental Section

Materials

Peptide cyclo-(Trp-Tyr) (cyclo-WY) was purchased from Bachem Company. Sodium alginate (ALG), hyaluronic acid (HA), poly-L-Lysine (PLL), nile red (NR) and thioflavin T (ThT) were obtained from Sigma Aldrich Chemical Company. Sodium hydroxide (NaOH), hydrochloric acid (HCl), dimethyl sulfoxide (DMSO) were products of Beijing Chemical Co. Ltd. All solutions were freshly prepared for immediate use. Water was prepared in a double-stage Milipore Milli-Q Plus purification system.

Methods

Preparation of the hydrogel

The cyclo-WY hydrogel was prepared through a solvent replacement method. Typically, a DMSO solution (20 μL) of cyclo-WY (2 mg) was prepared by heating in a water bath at 55 °C until completely dissolved. The DMSO solution of cyclo-WY was mixed with 480 μL pure water and the obtained mixture was aged for 48 h at room temperature. Hydrogel was formed. The hydrogel was repeatedly washed by water and was freeze-dried for further characterizations.

Characterization

Fourier transform infrared spectroscopy (FTIR) spectra were obtained by a spectrometer (TENSOR-27, Bruker). Scanning electron microscopy (SEM) images were taken with an S-4800 scanning electron microscope, as follows: A thin layer of

hydrogel was dropped on a 5 mm × 5 mm silicon slice and dried in a vacuum system. Samples were coated with Au on a BAL-TEC (SCD 050 sputter) before imaging. Transmission electron microscopy (TEM) images were obtained using a JEM-1011 microscope (JEOL). Confocal laser scanning microscope (CLSM) images were obtained by an Olympus FV500 microscope. X-ray diffraction (XRD) patterns were performed on an Empyrean instrument (Panalytical, Netherlands). A polarizing Optical Microscope (POM, Olympus BX53) was utilized to study the polarization properties of the hydrogels. Thermogravimetric analysis (TGA) results were recorded on a thermal analysis instrument (Q600 Simultaneous DSC-TGA). The samples were heated from 25 °C to 400 °C at a constant rate of 10 °C min^{-1} under N_2 atmosphere of 50 mL min^{-1}. The rheological properties of hydrogels were tested using a rotational rheometer (Anton paar MCR302) at 25 °C , as follows: the hydrogel samples were placed on the middle of a 15 mm diameter parallel plate. Preventing the evaporation of water, a lid was used. Dynamic oscillatory strain amplitude sweep measurements were studied at a frequency of 10 rad s^{-1}. Dynamic oscillatory frequency sweep measurements were conducted at 1% strain amplitude. An alternation of larger strain of 500% and small strain of 0.1% was applied on hydrogels for several cycles, to study the recovery behavior of hydrogels.

Stability of hydrogels

Charged biopolymers, including positive charged PLL and negative charged HA and ALG, were used. The composite hydrogels were typically prepared as follows: 20 μL DMSO solution of cyclo-WY (2 mg) was mixed with 480 μL water solution of

biopolymer (PLL or HA or ALG, 0.1 mg). The morphology, rheological properties and XRD patterns were conducted utilizing SEM, rotational rheometer and XRD instruments. Hydrogels of pH 1 and pH 14 were prepared as follows: 20 uL DMSO solution of cyclo-WY (2 mg) was heated in a water bath at 55 ℃ until completely dissolved; the DMSO solution of cyclo-WY was mixed with 480 μL pure water and then pH of the final samples were adjusted to 1 or 14. The hydrogels were aged for 48 h before further characterization.

Electrochemical measurements

The electrochemical behavior of hydrogels were studied on a glassy carbon (GC) electrode. The electrode was preliminary polished with 0.05 μm alumina slurries and coated with a layer of the hydrogel (10 μL). The electrode was subsequently air-dried and tested on a CHI 660E electrochemical work station.

Additional Figures

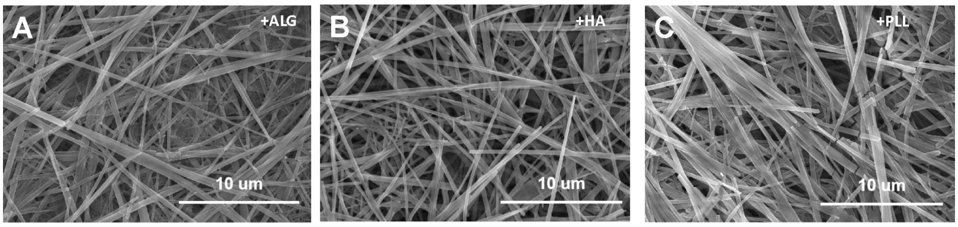

Figure S1 SEM photos of cyclo-WY/biopolymer hydrogels.

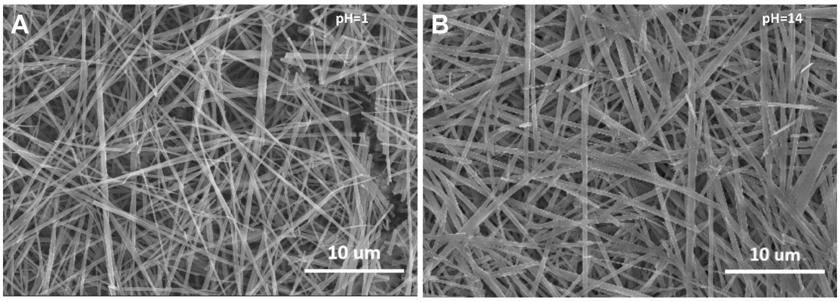

Figure S2 SEM images of cyclo-WY hydrogel at different pH values.

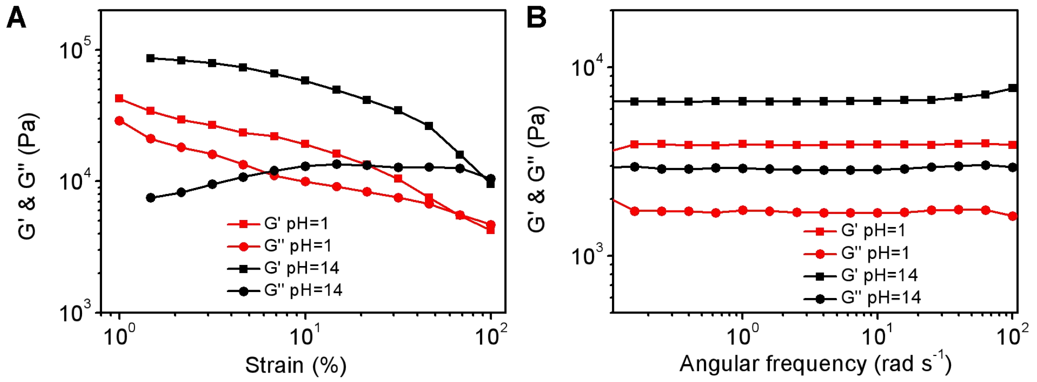

Figure S3 Strain-dependent (A) and (B) frequency-dependent oscillatory shear rheology of the hydrogels at pH 1 and pH 14.
